# Hospital Costs and Pleural Complications of Electromagnetic Navigational Bronchoscopy Versus CT-Guided Transthoracic Needle Biopsy: A German Multicentre Matched Analysis

**DOI:** 10.3390/jcm15218648

**Published:** 2026-11-06

**Authors:** Jacopo Saccomanno, Martin Witzenrath, Nikolaj Frost, Ralf Hammerich, Thomas Elgeti, Susanne Lang, Carolin Kraska, Dominic Dellweg, Patrick Parschke, Daniel Drömann, Goran Mitkovski, Konrad Wetzer, Olaf Schega, Stephanie Burian, Stefan Morgenstern, Burkhard Bewig, Karina Kleinschnitz, Wolfgang Heinlein, Markus Rathmayer, Ralf-Harto Hübner

**Affiliations:** 1 Department of Infectious Diseases, Respiratory Medicine and Critical Care, Charité–Universitätsmedizin Berlin, Corporate Member of Freie Universität Berlin and Humboldt-Universität zu Berlin, Campus Benjamin Franklin, Hindenburgdamm 30, 12203 Berlin, Germany; 2 German Center for Lung Research (DZL), 10117 Berlin, Germany; 3 Capnetz Foundation, 30625 Hannover, Germany; 4 Administrative Office for Clinical Quality and Risk Management, Charité–Universitätsmedizin Berlin, 10117 Berlin, Germany; 5 Department of Radiology, Charité–Universitätsmedizin Berlin, Corporate Member of Freie Universität Berlin and Humboldt-Universität zu Berlin, 12203 Berlin, Germany; 6 Medizinische Klinik V, Universitätsklinikum Jena, 07747 Jena, Germany; 7 Klinik für Innere Medizin, Pneumologie und Gastroenterologie, Pius-Hospital Oldenburg, 26121 Oldenburg, Germany; 8 Medizinische Klinik III, Universitätsklinikum Schleswig-Holstein (UKSH), 23538 Lübeck, Germany; 9 Airway Research Center North (ARCN), German Center for Lung Research (DZL), 23538 Lübeck, Germany; 10 Klinik für Pneumologie und Intensivmedizin, Klinikum St. Georg Leipzig, 04129 Leipzig, Germany; 11 Fachklinik Pneumologie, Thoraxchirurgie und Allergologie, Johanniterkrankenhaus Treuenbrietzen, 14929 Treuenbrietzen, Germany; 12 Klinik für Pneumologie, Infektiologie und Internistische Intensivmedizin, Klinikum Dortmund, 44145 Dortmund, Germany; 13 Klinik für Pneumologie, Allergologie, Schlaf- und Beatmungsmedizin, Evangelische Kliniken Essen-Mitte gGmbH, 45276 Essen, Germany; 14 Medizinische Klinik, Städtisches Krankenhaus Kiel, 24116 Kiel, Germany; 15 Inspiring-Health GmbH, 80935 Munich, Germany

**Keywords:** bronchoscopy, electromagnetic navigation, hospital costs, lung biopsy, pneumothorax, pulmonary nodules, transthoracic needle biopsy

## Abstract

**Background/Objectives:** Electromagnetic navigational bronchoscopy (ENB) and CT-guided transthoracic needle biopsy (TTNB) are established approaches to sampling peripheral pulmonary lesions. Comparative real-world data on procedure-associated pleural complications and hospital resource use remain limited. We compared coded pleural complications, length of stay, hospital costs and reimbursement after ENB versus TTNB in German inpatient care. **Methods:** This retrospective multicentre analysis used fully anonymised administrative hospital cost and reimbursement data from nine German hospitals between January 2023 and December 2024. Of 2904 inpatient cases (590 ENB and 2314 TTNB), 536 matched pairs were generated by 1:1 nearest-neighbour propensity-score matching without replacement using age and sex, with exact matching on adjacent diagnosis-related group (ADRG2). Outcomes were coded pneumothorax, coded pneumothorax requiring chest-tube drainage, length of stay, total hospital costs and reimbursement. **Results:** In the matched cohort, mean age was 68.2 ± 10.6 years after TTNB and 68.5 ± 10.4 years after ENB; the male-to-female ratio was 1.34 and 1.36, respectively. Coded pneumothorax occurred less frequently after ENB than after TTNB (4.1% vs. 13.1%); in matched-pair logistic regression, ENB was associated with lower odds of coded pneumothorax (OR 0.28, 95% CI 0.17–0.47; *p* < 0.001). Coded pneumothorax requiring chest-tube drainage occurred in 3.4% versus 12.9%, respectively. Median length of stay was shorter after ENB (2 [IQR 1–4] vs. 3 [1–8] days; *p* < 0.001), whereas median hospital costs were higher (€5793 [4670–7466] vs. €3739 [1995–7514]; *p* < 0.001). Median reimbursement was €3366 in both groups (*p* = 0.186). **Conclusions:** In this German inpatient cohort, ENB was associated with fewer coded pleural complications and shorter hospital stays than TTNB, but with higher hospital costs despite similar reimbursement. These findings characterise system-specific inpatient resource use and do not establish comparative clinical superiority or cost-effectiveness.

## 1. Introduction

Pulmonary nodules are increasingly detected through incidental imaging and lung cancer screening, increasing the demand for tissue diagnosis when histological confirmation is indicated [1,2,3,4,5]. Procedure selection must balance diagnostic performance and tissue adequacy against procedural risk and resource use. CT-guided transthoracic needle biopsy (TTNB) provides direct percutaneous access to peripheral pulmonary lesions, but transpleural needle passage carries a clinically relevant risk of pneumothorax, occasionally requiring chest-tube drainage or hospitalisation [6,7].

Guided bronchoscopy offers an endobronchial route to peripheral lesions and may combine electromagnetic navigation with radial endobronchial ultrasound, fluoroscopy, digital tomosynthesis or cone-beam CT (CBCT) [8,9,10,11]. Accordingly, the clinically relevant distinction is bronchoscopic versus transthoracic access rather than simply “CT-guided” versus bronchoscopic biopsy. In the VERITAS randomised trial, navigational bronchoscopy achieved non-inferior diagnostic accuracy compared with TTNB and was associated with fewer pneumothoraces [12]. The 2025 AABIP evidence-based guideline similarly addresses diagnostic yield and complications of peripheral bronchoscopy versus TTNB and emphasises lesion characteristics, evolving technology, local expertise and available resources in procedural decision-making [13].

Procedure selection also depends on the ability to obtain adequate tissue for molecular testing. The 2025 AABIP/IASLC guideline recommends guided bronchoscopy as the safer approach for accessible parenchymal lesions and considers bronchoscopic and percutaneous sampling comparable for comprehensive biomarker testing, while emphasising patient-, lesion- and centre-specific factors [14]. These clinical dimensions are not captured by administrative reimbursement data and therefore cannot be inferred from hospital cost comparisons.

Contemporary comparative studies increasingly define diagnostic performance and procedural safety of ENB and TTNB, but real-world inpatient resource use, hospital costs and reimbursement within the German G-DRG system remain poorly characterised. Previous economic studies have largely relied on modelling or cost-consequence estimates [15,16]. We therefore compared coded pleural complications, length of stay, hospital costs and reimbursement after ENB and CT-guided TTNB across nine German hospitals. The analysis was restricted to the hospital perspective and was not designed to assess overall diagnostic effectiveness, clinical superiority or cost-effectiveness.

## 2. Materials and Methods

### 2.1. Study Design and Setting

This retrospective multicentre study used fully anonymised routine hospital cost and reimbursement data from nine German hospitals participating in the InEK cost-accounting framework. Eligible inpatient cases underwent ENB or CT-guided TTNB between January 2023 and December 2024. Hospital identifiers were removed before data transfer and were not available for analysis. Analyses were conducted from the perspective of the treating hospital within the German Diagnosis-Related Group (G-DRG) system. InEK hospitals contribute standardised case and cost data used to calculate and refine national inpatient reimbursement [17,18].

### 2.2. Exposure Definitions and Procedural Scope

CT-guided TTNB was defined as percutaneous tissue sampling through the chest wall under CT-based image guidance. We use the term TTNB because CT- or CBCT-based imaging may also support modern guided bronchoscopy [11,14]. ENB denotes electromagnetic navigational bronchoscopy as coded by the participating hospitals. The administrative dataset did not permit standardised reconstruction of procedure-level adjuncts across centres, including radial EBUS, fluoroscopy or tomosynthesis, CBCT confirmation, anaesthesia strategy and biopsy-tool selection. ENB should therefore be interpreted as a routine-care bronchoscopic pathway rather than a uniform technical protocol.

### 2.3. Study Population and Matching

Inclusion criteria were inpatient ENB or CT-guided TTNB between January 2023 and December 2024 and available hospital cost data. For the matched analysis, cases additionally required non-missing age, sex and ADRG2. Cases not meeting these requirements were excluded from the corresponding analysis; no additional lesion-level clinical exclusion criteria were applied because these variables were unavailable in the administrative dataset. The unmatched cohort comprised 2904 cases: 590 ENB procedures and 2314 CT-guided TTNB procedures. No a priori sample-size calculation was performed because all eligible cases were included. Propensity scores were estimated using age and sex. Patients were then matched 1:1 by nearest-neighbour matching without replacement, with exact matching on ADRG2, yielding 536 matched pairs. Age and sex were selected as consistently available demographic covariates, while exact ADRG2 matching constrained comparisons to reimbursement-relevant case-mix groups. Absolute standardised mean differences (SMDs) for age and sex were 0.031 and 0.030 before matching and 0.028 and 0.008 after matching, respectively, indicating excellent post-matching balance. ADRG2 (Adjacent Diagnosis-Related Group; base DRG) is a higher-level grouping within the German G-DRG classification [17]. It does not encode lesion size, lesion location, bronchus sign, pleural distance or other determinants of biopsy selection. Pulmonary function, emphysema, procedure-relevant comorbidities, operator experience and centre volume were likewise unavailable. The matched analysis therefore reduces imbalance only in observed administrative variables and cannot eliminate confounding by indication or establish clinical equivalence between groups.

### 2.4. Outcome Definitions and Economic Perspective

Outcomes were coded pneumothorax, coded pneumothorax requiring chest-tube drainage, length of stay, total hospital costs, reimbursement and the cost-reimbursement gap. Complications were identified from administrative coding within the G-DRG dataset; minor or self-limited radiographic pneumothoraces were not prospectively adjudicated. Diagnostic yield, diagnostic accuracy, biomarker adequacy, repeat biopsy, time to diagnosis, downstream oncological treatment, patient-reported morbidity and quality-adjusted life years (QALYs) were unavailable. The economic analysis therefore describes hospital resource use and cost consequences in German inpatient care and is not a formal cost-effectiveness or cost-utility analysis.

### 2.5. Statistical Analysis

Continuous variables were summarised as mean ± SD or median (IQR), as appropriate, and categorical variables as counts and percentages. In the unmatched cohort, age was compared using Student’s *t*-test and other continuous variables using Wilcoxon rank-sum tests. In the matched cohort, binary outcomes were analysed using logistic regression with cluster-robust standard errors and continuous outcomes using linear regression with cluster-robust standard errors, with clustering by matched pair. The primary models therefore account for matched-pair dependence but do not explicitly model hospital-level clustering. Hospital-level sensitivity modelling was not possible because hospital identity had been irreversibly removed during anonymisation before data transfer; reconstructing hospital identity from site codes and case volumes could also create a risk of institutional re-identification. Results are reported as odds ratios (ORs) or regression coefficients with 95% confidence intervals (CIs). No imputation was performed. All tests were two-sided, with *p* < 0.05 considered statistically significant. Analyses were performed in R (R Foundation for Statistical Computing, Vienna, Austria).

### 2.6. Reporting and Ethics

The study was reported with reference to STROBE and the RECORD extension for studies using routinely collected health data [19,20]. Patients and the public were not involved because the study used fully anonymised administrative data. Ethical review was not required because the analysis contained no identifiable individual patient information and involved no intervention in diagnosis or treatment, in accordance with the German Model Professional Code for Physicians (MBO-Ä) and corresponding professional regulations [21].

## 3. Results

**Figure 1:**
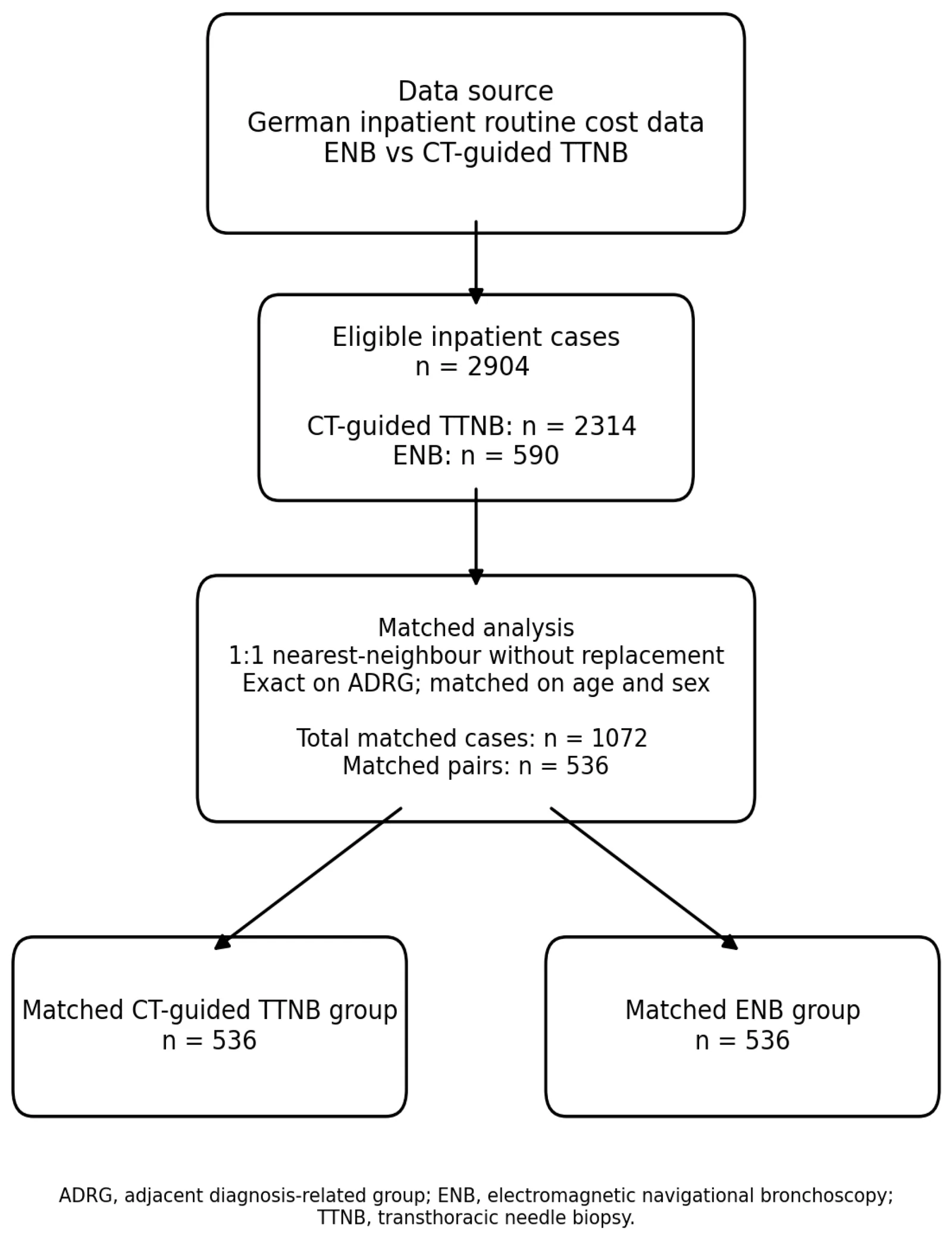
Study flow diagram of cohort selection and matching. Propensity-score matching was performed 1:1 using age and sex, with exact matching on ADRG2, yielding 536 matched pairs. ADRG2, adjacent diagnosis-related group; ENB, electromagnetic navigational bronchoscopy; TTNB, transthoracic needle biopsy.

Of 2904 eligible inpatient cases, 2314 underwent CT-guided TTNB and 590 underwent ENB. After 1:1 nearest-neighbour matching based on age and sex with exact matching on ADRG2, 1072 cases remained (536 matched pairs; Figure 1).

In the matched cohort, mean age was 68.2 ± 10.6 years after TTNB and 68.5 ± 10.4 years after ENB (*p* = 0.647). Men accounted for 57.3% (307/536) of the TTNB group and 57.6% (309/536) of the ENB group. Post-matching absolute SMDs were 0.028 for age and 0.008 for sex. Median length of stay was 3 (IQR 1–8) days after TTNB and 2 (1–4) days after ENB (*p* < 0.001). Median total hospital costs were €3739 (1995–7514) for TTNB and €5793 (4670–7466) for ENB (*p* < 0.001). Median reimbursement was €3366 in both groups (TTNB: 1470–4052; ENB: 1738–4052; *p* = 0.186), resulting in a larger median cost-reimbursement gap after ENB (€2516 [1409–4082] vs. €473 [−984–3368]; *p* < 0.001) (Table 1).

The most frequent final DRGs are shown in Table S1. Despite exact matching on ADRG, final DRG distributions remained heterogeneous, indicating residual differences in reimbursement-related case mix.

Coded pneumothorax occurred in 70/536 (13.1%) TTNB cases and 22/536 (4.1%) ENB cases (*p* < 0.001). Coded pneumothorax requiring chest-tube drainage occurred in 69/536 (12.9%) and 18/536 (3.4%) cases, respectively, corresponding to an absolute difference of 9.5 percentage points (*p* < 0.001) (Table 2).

In matched-pair logistic regression, ENB was associated with lower odds of coded pneumothorax than TTNB (OR 0.28, 95% CI 0.17–0.47; *p* < 0.001), whereas age and male sex were not associated with the outcome (Table 3). Because clinically relevant selection variables were unavailable, this estimate should be interpreted as an adjusted association within the observed administrative covariates rather than as a causal treatment effect.

In matched-pair linear regression, coded pneumothorax was associated with a 3.13-day longer hospital stay (95% CI 0.97–5.28; *p* = 0.005), whereas ENB was associated with a 2.28-day shorter stay (95% CI −3.05 to −1.51; *p* < 0.001) (Table 4). In the hospital-cost model, each additional hospital day was associated with €952.19 higher total costs (95% CI 728.79–1175.59; *p* < 0.001), while ENB remained associated with €3552.11 higher costs (95% CI 2399.44–4704.79; *p* < 0.001) (Table 5). Total hospital costs represent aggregate case-level inpatient costs rather than an itemised procedure-specific charge. Thus, costs associated with management of complications, including chest-tube drainage where applicable, are contained within the overall hospital episode but cannot be isolated as a distinct component. The €952.19 coefficient represents an adjusted association within the observed data and should not be interpreted as a fixed per-day tariff or as the specific cost of chest-tube drainage.

For descriptive context, the 9.5-percentage-point absolute difference in coded chest-tube pneumothorax corresponds to approximately one fewer event per 11 ENB procedures. With an additional mean hospital cost of €1016 per ENB case, this equates to approximately €10,675 in additional hospital expenditure per one fewer coded chest-tube pneumothorax (Table S2). This is a descriptive cost-consequence metric; it is neither a causal number needed to treat nor a cost-effectiveness ratio and does not incorporate diagnostic performance, QALYs or downstream care.

## 4. Discussion

Three findings characterise this multicentre analysis of German inpatient administrative data. First, ENB was associated with fewer coded pneumothoraces, including fewer events requiring chest-tube drainage. Second, ENB was associated with a shorter hospital stay. Third, these differences did not translate into lower hospital costs: ENB remained more costly, while reimbursement was similar between groups. The study therefore describes a system-specific trade-off in inpatient resource use rather than comparative clinical superiority or cost-effectiveness.

The lower rate of coded pleural complications with ENB is consistent with contemporary comparative evidence. VERITAS reported non-inferior diagnostic accuracy for navigational bronchoscopy versus TTNB with fewer pneumothoraces [12], and a systematic review similarly found a favourable safety profile for navigation bronchoscopy [22]. Procedure selection in clinical practice depends on lesion size and location, bronchus sign, pleural distance, patient comorbidity, staging requirements, expected diagnostic yield, local expertise and available technology [13,14]. These variables were unavailable in our dataset; therefore, our results cannot define lesion-specific selection criteria or identify patients who should preferentially undergo ENB or TTNB.

The economic findings require equally cautious interpretation. ENB was associated with shorter hospitalisation but higher total hospital costs, and the cost difference persisted after accounting for length of stay. Within this inpatient dataset, shorter stays therefore did not offset the higher costs associated with the ENB pathway. The model cannot attribute this residual difference to specific procedural cost components. Previous studies have likewise reported higher upfront costs for ENB alongside fewer complications [15,16]. The economic findings are specific to German inpatient care within the G-DRG/InEK cost-accounting framework. Hospital admission practices, procedure costs, tariffs and reimbursement mechanisms differ across healthcare systems; consequently, the absolute cost and reimbursement estimates should not be extrapolated directly to other countries or to ambulatory settings. Moreover, the absence of diagnostic yield, repeat procedures, biomarker adequacy, downstream treatment, patient-reported outcomes and QALYs precludes a comprehensive economic evaluation.

The principal methodological limitation is residual confounding by indication. Matching on age, sex and ADRG2 achieved excellent balance for the observed demographic covariates but cannot account for lesion size or location, bronchus sign, pleural distance, emphysema, pulmonary function, procedure-relevant comorbidities, operator experience or centre volume. The primary models cluster by matched pair and do not account for hospital-level clustering. Hospital identifiers were intentionally removed during anonymisation and were unavailable for analysis; reconstruction from case volumes could permit institutional re-identification. A hospital-level sensitivity analysis was therefore not possible. Multicentre prospective ENB studies have reported low complication rates across broad clinical implementation [8,23,24], supporting reproducibility of the technique; nevertheless, centre-level heterogeneity in patient selection, procedural pathways, coding, technology and operator experience cannot be excluded in our dataset.

## 5. Conclusions

In German inpatient care, ENB was associated with fewer coded pleural complications and shorter hospital stays than CT-guided TTNB, but with higher hospital costs and similar reimbursement. These findings identify a resource-use trade-off within a specific reimbursement environment; they do not establish clinical superiority or cost-effectiveness. Procedure selection should remain based on patient and lesion characteristics, expected diagnostic yield and tissue adequacy, procedural risk, staging requirements and local expertise. Future prospective comparative studies should integrate lesion-level clinical determinants, operator and centre characteristics, diagnostic performance, biomarker adequacy, downstream care and ambulatory pathways.

## Figures and Tables

**Table 1:** Matched cohort characteristics and hospital resource use.

Variable	CT-Guided TTNB	ENB	*p* Value
Procedures (*n*)	536	536	—
Male sex, n (%)	307 (57.3%)	309 (57.6%)	—
Age (years)	68.2 ± 10.6	68.5 ± 10.4	0.647
Length of stay (days)	3 (1–8)	2 (1–4)	<0.001
Total hospital costs (€)	3739 (1995–7514)	5793 (4670–7466)	<0.001
Hospital reimbursement (€)	3366 (1470–4052)	3366 (1738–4052)	0.186
Cost-reimbursement gap (€)	473 (−984–3368)	2516 (1409–4082)	<0.001

Data are mean ± SD, median (IQR), or n (%). TTNB, transthoracic needle biopsy; ENB, electromagnetic navigational bronchoscopy; SMD, standardised mean difference. Absolute SMDs for age and sex were 0.031 and 0.030 before matching and 0.028 and 0.008 after matching, respectively. The cost-reimbursement gap represents case-level hospital costs relative to reimbursement and is system-specific.

**Table 2:** Coded pleural complications in the matched cohort.

Outcome	CT-Guided TTNB	ENB	*p* Value
No coded pneumothorax	466 (86.9%)	514 (95.9%)	<0.001
Coded pneumothorax	70 (13.1%)	22 (4.1%)	<0.001
Coded pneumothorax requiring chest-tube drainage	69 (12.9%)	18 (3.4%)	<0.001

Events were identified from administrative inpatient coding. Minor or self-limited radiographic pneumothoraces may be under-recorded. Abbreviations: ENB, electromagnetic navigational bronchoscopy; TTNB, transthoracic needle biopsy.

**Table 3:** Matched-pair logistic regression for coded pneumothorax.

Variable	Odds Ratio (95% CI)	*p* Value
Procedure (ENB vs. TTNB)	0.28 (0.17–0.47)	<0.001
Age	1.01 (0.99–1.03)	0.177
Male sex	0.99 (0.64–1.53)	0.959

Cluster-robust standard errors account for matched pairs. The model does not adjust for unavailable clinical selection factors. Abbreviations: ENB, electromagnetic navigational bronchoscopy; TTNB, transthoracic needle biopsy; OR, odds ratio; CI, confidence interval.

**Table 4:** Matched-pair linear regression for length of stay.

Variable	B Coefficient (95% CI)	*p* Value
Coded pneumothorax	3.13 (0.97–5.28)	0.005
Procedure (ENB vs. TTNB)	−2.28 (−3.05 to −1.51)	<0.001
Age	0.04 (−0.01–0.09)	0.085
Male sex	0.10 (−0.85–1.05)	0.840

Cluster-robust standard errors account for matched pairs. Abbreviations: ENB, electromagnetic navigational bronchoscopy; TTNB, transthoracic needle biopsy; B, regression coefficient; CI, confidence interval.

**Table 5:** Matched-pair linear regression for total hospital costs.

Variable	B Coefficient (95% CI)	*p* Value
Length of stay (days)	952.19 (728.79–1175.59)	<0.001
Procedure (ENB vs. TTNB)	3552.11 (2399.44–4704.79)	<0.001
Age	3.92 (−26.96–34.80)	0.804
Coded pneumothorax	−921.07 (−1943.43–101.29)	0.078
Male sex	−36.91 (−970.50–896.68)	0.938

Cluster-robust standard errors account for matched pairs. Abbreviations: ENB, electromagnetic navigational bronchoscopy; TTNB, transthoracic needle biopsy; B, regression coefficient; CI, confidence interval.

## Data Availability

Data may be made available on reasonable request to the corresponding author, subject to applicable data-use agreements with participating hospitals.

## Supplementary Materials

The following supporting information can be downloaded at https://www.mdpi.com/article/10.3390/jcm15218648/s1, Table S1: Top 10 final DRG distribution in the matched cohort; Table S2: Descriptive hospital cost-consequence calculation based on the observed chest-tube pneumothorax difference.
