# Supplementary material for: Hospital Costs and Pleural Complications of Electromagnetic Navigational Bronchoscopy Versus CT-Guided Transthoracic Needle Biopsy: A German Multicentre Matched Analysis
Source: J Clin Med. 2026 Nov 6;15(21):8648. doi: 10.3390/jcm15218648 (PMC13763806; doi:10.3390/jcm15218648)
Supplement: Supplementary file 1 [file jcm-15-08648-s001.zip › jcm-4620714-supplementary.pdf]

Supplementary Materials

Electromagnetic Navigational Bronchoscopy versus CT-Guided Transthoracic Needle Biopsy: A German Multicentre Matched Analysis

Table S1. Top 10 Final DRG Distribution in the Matched Cohort

DRG	Description	CT-guided TTNB n (%)	ENB n (%)
E71C	Respiratory neoplasms; low complexity; rigid bronchoscopy or percutaneous respiratory biopsy	256 (47.8%)	167 (31.2%)
E02D	Other respiratory OR procedures; adults; >1 day; no major laryngeal/tracheal procedure	64 (11.9%)	76 (14.2%)
E71B	Respiratory neoplasms; low complexity; esophageal prosthesis or endoscopic respiratory biopsy with chemotherapy	9 (1.7%)	103 (19.2%)
E02E	Other respiratory OR procedures; adults; low complexity; same-day stay	26 (4.9%)	43 (8.0%)
E71D	Respiratory neoplasms; low complexity; without rigid bronchoscopy or percutaneous respiratory biopsy	17 (3.2%)	17 (3.2%)
E69C	Bronchitis/asthma or respiratory symptoms; low complexity	16 (3.0%)	16 (3.0%)
E74Z	Interstitial lung disease	13 (2.4%)	13 (2.4%)
E02B	Other respiratory OR procedures; moderate complexity	19 (3.5%)	6 (1.1%)
E06D	Other lung resections / thoracic procedures	11 (2.1%)	12 (2.2%)
E02C	Other respiratory OR procedures; >1 day; selected moderate-complexity interventions	18 (3.4%)	4 (0.7%)
Other	All remaining DRGs outside the top 10	87 (16.2%)	79 (14.7%)

DRG descriptions are concise English summaries for readability; official German G-DRG wording is more detailed. TTNB, transthoracic needle biopsy; ENB, electromagnetic navigational bronchoscopy.

Table S2. Descriptive Hospital Cost-Consequence Calculation Based on the Observed Chest-Tube Pneumothorax Difference

Parameter	Result
Observed absolute difference in coded chest-tube pneumothorax	9.5 percentage points
ENB procedures corresponding to one fewer coded chest-tube pneumothorax	≈11
Additional mean hospital cost of ENB versus CT-guided TTNB	€1,016
Estimated additional hospital cost associated with a coded chest-tube pneumothorax	€5,575
Incremental hospital expenditure per one fewer coded chest-tube pneumothorax	€10,675

This descriptive calculation is based on observed inpatient event and cost differences. It is not a cost-effectiveness or cost-utility ratio and does not incorporate diagnostic yield, biomarker adequacy, QALYs, repeat procedures, or downstream care.
